# Mesenchymal Stem Cells Senescence: Mechanism and Rejuvenation Interventions

**DOI:** 10.7150/ijms.115650

**Published:** 2025-07-28

**Authors:** Qinghong Meng, Yan Zheng, Xiaobo Chen, Jiawei Mu, Changlin Li, Yiwen Gao, Wei Liu, Yuliang Wang

**Affiliations:** 1 Department of Clinical Laboratory Medicine, The Eco-city Hospital of Tianjin Fifth Central Hospital, Tianjin, China.; 2 Clinical Laboratory, Wanxin Street Community Healthcare Center, Tianjin, China.; 3 Unicell Life Science Development Co., Ltd, Tianjin, China.; 4 The Second Hospital of Tianjin Medical University, Tianjin Institute of Urology, Tianjin, China.; 5 Tianjin Children's Hospital, Tianjin, China.; 6 Children's Hospital, Tianjin University, Tianjin, China.

**Keywords:** mesenchymal stromal/stem cells, ageing, cellular senescence, priming interventions

## Abstract

Aging has become one of the most significant challenges and burdens on public health and healthcare systems worldwide. However, it is possible to slow down the aging process through various interventions. Mesenchymal stromal/stem cells (MSCs) have emerged as one of the most promising therapeutic agents for combating aging and treating various age-related chronic medical conditions. This is primarily due to their well-known cellular plasticity and potent multipotency, which enable them to promote tissue repair and regeneration, as well as address inflammatory conditions. Remarkably, the high quality and functional activity of MSCs are negatively affected by cellular senescence, particularly in both healthy-aging MSCs and replicative senescent MSCs. This is a critical issue when considering the provision of "personalized" or "universal" clinical-grade products. Therefore, this review aims to summarize the biological properties, immunomodulatory dysfunction, and underlying mechanisms of senescent MSCs. Additionally, it discusses the current promising techniques published for rejuvenating senescent MSCs and optimizing their therapeutic potential.

## 1. Introduction

Mesenchymal stromal/stem cells (MSCs), as multipotent mesoderm-derived progenitor cells, can promote tissue repair and regeneration, and alleviate organ dysfunction 1. Human MSCs are at the forefront of medical research and are being evaluated as therapeutic agents in multiple clinical trials. There are currently almost 1,500 MSC-based clinical trials registered at clinicaltrials.gov database 2, ranging from Phase I to III, with many either completed or currently ongoing. Accumulating clinical evidence highlights the efficacy and safety of MSCs in treating severe COVID-19 and acute respiratory distress syndrome 3. Based on these findings, we propose the term of “*MSCs* +” (a rhetorical device). This term leverages the pluripotent and paracrine factors of MSCs (e.g., secretome, microvesicles, or exosomes) to achieve immunomodulation, anti-inflammatory, antiviral, anti-apoptotic, anti-fibrotic, and regenerative effects, offering potent therapeutic potential for various clinical conditions 4,5.

Ageing, a natural, multifactorial, and degenerative process, influences socioeconomic and public policies over time, representing a significant challenge 6. According to the National Bureau of Statistics of China, the population aged 65 and above exceeded 200 million, accounting for 14.9% of the total population by the end of 2022. This figure is expected to surpass 400 million, representing more than 30% of the population by 2050, marking the onset of severe aging in China 7. Additionally, World Health Organization predicts that one in six people worldwide will be over 65 by 2050 8. This indicates that a globally aging society will pose a significant challenge to the healthcare system and humanity, raising a foreseeable concern. MSCs are increasingly recognized as viable and ideal therapeutic agents for combating the effects of aging and treating various age-related diseases in the elderly, such as malignancies, cardiovascular conditions, skeletal disorders, immune/inflammatory diseases, and cognitive impairments 9. However, despite successful clinical applications, we cannot overlook the senescence-related changes in MSCs, which are natural responses to aging and accumulated stress from excessive cell proliferation. These changes reduce regenerative potential and undermine the therapeutic efficacy of MSCs in clinical trials. On one hand, although autologous MSCs are favored over allogeneic ones, their function declines with donor age, leading to senescence and reduced therapeutic efficacy 10. On the other hand, MSCs isolated from young donors require extensive monolayer culture and in vitro expansion to achieve sufficient quantities for clinical use. However, this process induces replicative senescence (*in vitro* aging), with each generation leading to a loss of stemness. This includes changes in intrinsic MSC characteristics, such as pluripotency, immunomodulatory properties, and paracrine function—inevitable aspects of cell manufacturing 11.

The acquisition of high-quality MSCs is currently considered one of the biggest technical challenges, particularly in preventing their premature senescence *in vitro* or *in vivo* during translation to clinical practice 12. To address these challenges, it is crucial to understand the features of senescence and develop specific strategies to rejuvenate senescent MSCs, aiming to generate a large number of youthful and viable MSCs for cell-based therapies. This review summarizes the biological properties, immunomodulatory dysfunctions, and driving mechanisms of senescent MSCs, while discussing promising techniques for rejuvenating these cells and optimizing their therapeutic potential.

## 2. Identification of MSC Senescence

As MSCs undergo senescence, their characteristics change significantly, including alterations in cellular morphology, phenotype, cell cycle, intracellular senescence-associated β-galactosidase (SA-β-gal) activity, senescence-related genes, the senescence-associated secretory phenotype, and intracellular adenosine triphosphate levels 13.

### 2.1 Morphological changes

**Figure 1:**
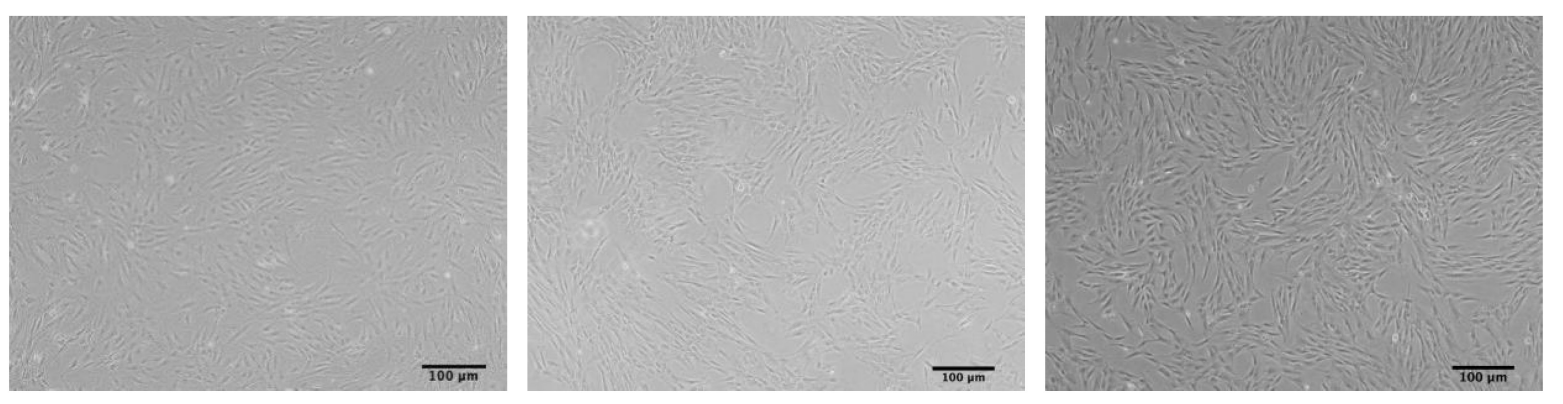
The morphology of ADSCs. ADSCs at passage 3 (left), ADSCs at passage 10 (middle) and ADSCs at passage 12 (“fried egg” shape) (right) (scale bars: 100 μm).

MSCs typically exhibit an elongated fibroblast like morphology when cultured under standard conditions. However, MSCs derived from late passages *in vitro* or from elderly donors, particularly those over 70 years old, often exhibit a noticeable morphological change, becoming flattened and enlarged, resembling a "fried egg" with constricted nuclei and granular cytoplasm 14-16. This alteration in cell size and morphology serves as a hallmark for evaluating cellular senescence, as exhibited in **Figure 1** 17. Furthermore, nuclear morphometric analysis (NMA), based on quantitative parameters of geometric features such as cells, nuclei, or nucleoli, provides a valuable tool for monitoring MSC senescence 18. He et al. recently developed a Cascade region-based convolutional neural network (Cascade R-CNN) system to detect both replicative and drug-induced senescent MSCs by identifying single cells of various sizes and shapes in multicellular images, providing a real-time platform for morphological image analysis 19.

### 2.2 Cell phenotypic changes

#### 2.2.1 Specific surface markers

According to the guidelines of the International Society for Cellular Therapy (ISCT), cultured MSCs are identified by the positive expression of cell surface markers CD73, CD90, and CD105, and the absence of endothelial and hematopoietic markers such as CD45 and CD34. Evidence indicates that the expression levels of these positive surface markers are downregulated with donor age or prolonged culture. For instance, Truong et al. 20 observed that the expression of CD105 dropped from 92.43% in early-passaged (p5) to 48.87% in late-passaged (p15) MSCs during *in vitro* culture. In another study, Martini et al. 21 suggested that the decrease in CD90 expression could serve as a distinct marker for the aging of cardiac-derived MSCs (cMSCs).

#### 2.2.2 Other surface markers

Additionally, evidence suggests that other surface marker expression profiles play an important role in identifying senescent MSCs. For instance, CD106 (vascular cell adhesion molecule-1) and CD228 (melanotransferrin) were significantly downregulated in late-passaged (p14) and (p16-18) MSCs, respectively, and were associated with reduced cell adhesion, migration, and multi-differentiation capabilities 22,23. It has been reported that a decrease in CD146 expression accelerates cellular senescence by enhancing the expression of p53, p16, and p21, while inhibiting Bmi-1 expression in senescent MSCs. Additionally, several studies have observed an increase in senescence-related surface markers, such as CD264 and CD26, in MSCs, suggesting that these markers could serve as indicators of MSC quality. For example, MSCs showed a gradual increase in CD264 expression, accompanied by co-expression of p53 and p21 during subculture expansion, which strongly negatively correlated with their proliferation and multi-differentiation capabilities 24. Several studies have shown that CD26 expression is upregulated during the MSC senescence process both *in vitro* and *in vivo*, along with an increase in the expression of cell cycle arrest genes such as p16 and p21. This suggests that CD26 could serve as a senescence marker for human MSCs. Furthermore, CD26^high^ MSCs were less effective at suppressing T cell proliferation compared to CD26^low^ MSCs 25,26. Notably, inhibiting CD26 could reverse senescence-associated gene expression changes (p53 and p21), increase MSC surface adhesion, and enhance therapeutic effects in mouse lung emphysema 26. The surface phenotype mentioned above are listed in Table 1.

### 2.3 Intracellular SA-β-gal and senescence-related genes

#### 2.3.1 SA-β-gal and senescence-related genes

A well-established method for evaluating senescent cells and the senescence process is the detection of elevated intracellular senescence-associated β-galactosidase (SA-β-gal) activity 29. In addition to SA-β-gal, increased expression of p16, p21, and p53 are also recognized markers of cellular senescence. Previous research has shown increased SA-β-gal activity, upregulated p16 and p21 gene expression, and cell cycle arrest in late-passaged (p9~p14) human bone marrow-derived MSCs (BM-MSCs), (p18) umbilical cord blood-derived MSCs (UCB-MSCs), and (p10) adipose-derived MSCs (ADSCs) 30-32. It is worth noting that SA-β-gal activity is expressed by GLB1, the gene encoding lysosomal beta-D-galactosidase. When MSCs were genetically deficient in the GLB1 gene or completely lacked lysosomal β-galactosidase, aged MSCs did not express SA-β-gal 33.

#### 2.3.2 α-fuc

Recently, researchers identified α-l-fucosidase (α-fuc) as a biomarker for cellular senescence and developed an α-fuc aggregation-induced emission (AIE) probe, QM-NHαfuc. This probe enabled real-time tracking of senescent cells that lack β-galactosidase expression, both *in vitro* and *in vivo*, overcoming the limitations of traditional markers like SA-β-gal 34.

### 2.4 Senescence-associated secretory phenotype

#### 2.4.1 SASP production

When senescence occurs, MSCs also exhibit the senescence-associated secretory phenotype (SASP), which helps maintain cellular metabolism. This phenotype involves a variety of secretory proteins, including proinflammatory cytokines, chemokines, growth regulators, angiogenic factors, and matrix metalloproteinases (MMPs). These proteins are synthesized non-autonomously and secreted by senescent MSCs into the surrounding microenvironment through both autocrine and paracrine mechanisms. This process induces cells to exit the cell cycle and enter a state of cellular senescence via a regulatory network of cascades and feedback loops.

#### 2.4.2 The effect of SASP

The biologically active components of the SASP, such as interleukin (IL)-6, IL-8/CXCL8, and monocyte chemoattractant protein-1/CCL2 (MCP-1/CCL2), are released by MSCs from elderly donors into the microenvironment. These components play a role in evaluating the typical characteristics of senescent cells and can lead to negative health consequences. These include activating a cellular senescence program in early-passaged MSCs, triggering premature senescence in young MSCs, increasing inflammation and pathological changes in the bone marrow microenvironment, and contributing to a higher incidence of age-related osteoporosis 35. Alessio et al. observed certain components of insulin-like growth factor binding proteins (IGFBPs) in the SASP of senescent MSCs, and these IGFBPs could induce senescence in neighboring cells. Inactivating IGFBPs in the SASP abolished their pro-senescent effect 36. We compiled and compared the composition of the SASP released by different types of senescent MSCs (Table 2).

#### 2.4.3 Related signaling pathways

Notably, the composition of the SASP released by senescent MSCs may contribute to the activation of a cellular senescence program through various signaling pathways, each with distinct functions. These include: p38MAPK/MAPKAPK-2-mediated growth arrest by increasing NF-κB transcriptional activity 18; p53/p21/Rb- and p16/Rb-mediated cell growth arrest by inhibiting cyclin-dependent kinase (CDK), thus blocking E2F-mediated transcription of genes involved in cell proliferation 37; phosphatidylinositide 3-kinases (PI3K)/protein kinase B (PKB)/mammalian target of rapamycin (mTOR)-mediated autophagy, which synthesizes SASP factors through mTOR complex 1 bound to lysosomes 38; and ROS-prostaglandin signaling, which is mediated by internalization via caveolae and interaction with RARα 36. Currently, inhibitors or shRNA transfection targeting these relevant pathways are considered effective in suppressing SASP production, reducing inflammatory responses, and treating age-related diseases such as UVA-induced skin photoaging 39, degenerative disc disease 40, and osteoarthritis (OA) 41.

### 2.5 ATP and eccDNA

#### 2.5.1 ATP

Mitochondria are well-known cellular organelles responsible for maintaining cellular energy balance and metabolism through the production of adenosine triphosphate (ATP), a high-energy phosphate compound 45. However, aging is associated with cellular dysfunction and a decline in the energy required for cellular physiological functions, which directly impacts ATP availability 46. In a 2021 study by Herzig et al. 47, a reliable assessment of the immunosuppressive properties of MSCs on PBMCs was conducted using a luminescent ATP assay, which streamlined the analysis for comparing the potency of different MSC preparations. Therefore, we suggest that intracellular ATP could serve as an effective indicator in analyzing aging MSCs from donors or those undergoing replicative senescence.

#### 2.5.2 eccDNA

Furthermore, extrachromosomal circular DNA (eccDNA) is a form of double-stranded, closed, non-structured circular DNA that exists outside the chromosomes. Yang et al. 48 discovered that senescent BM-MSCs exhibited a distinct eccDNA landscape and expression pattern, and that a specific eccDNA could serve as a biomarker for BM-MSCs senescence.

### 3. Biological properties

Proliferative, migratory, anti-oxidative, and multilineage differentiation potentials are key biological properties of MSCs. However, MSCs gradually lose these properties as their proliferative and migratory capacities decline, oxidative stress resistance decreases, and differentiation becomes imbalanced, ultimately leading to senescence.

### 3.1 Proliferation, migration and anti-oxidation

MSCs senescence limits their clinical efficacy, primarily due to reduced proliferative and migratory capacities, as well as decreased tolerance to oxidative stress.

#### 3.1.1 Proliferation

Senescent MSCs are characterized by decreased levels of fibroblast colony-forming units (CFU-F) and increased population doubling time (PDT). More importantly, they exhibit a significant reduction in the number of cells in the S phase, along with a decline in their self-renewal capacity 15,31,49,50. After prolonged cell division during long-term in vitro culture, MSCs enter permanent G0 phase arrest, and mitosis is inhibited, although they remain viable. Several studies have shown that this process impairs the G1/G2-to-S phase and G2/M transitions of the cell cycle, upregulates cell cycle arrest-related genes such as p16, p21, and p27, and is primarily modulated through activation of the p53/p21WAF1/CIP1, Cyclin D1/CDK4/E2F1, and p16INK4A/Rb signaling pathways, resulting in cellular senescence 51,52.

#### 3.1.2 Migration

Given that migration and homing ability are key for MSCs in tissue repair and immunomodulation, previous studies have shown that with donor aging and cellular replicative senescence, the migration ability of MSCs is impaired, correlating with decreased levels of migration-related proteins such as chemokine receptors (CXCR4 and CXCR7) and MMP family members (MMP3, MMP9, MMP11, MMP13, and MMP14) 49,53,54. Similarly, our study demonstrated a significant inhibition of proliferation and migration potential in senescent MSCs, as detected by xCELLigence RTCA 17. Moreover, Amini-Nik et al. reported that senescence could affect MSCs migration to the wound bed, contributing to impaired regenerative capacities of MSCs in wound healing 55.

#### 3.1.3 Anti-oxidation

In addition to weakened proliferative and migratory capacities, the antioxidant ability of MSCs from elderly individuals was significantly reduced, as evidenced by lower activity of the antioxidant enzyme SOD and decreased resistance to hydrogen peroxide (H2O2) compared to MSCs from adults 56. More specifically, the expression levels of SOD1 to SOD3 genes in MSCs from elderly individuals were significantly downregulated 57. Compared to early-passage MSCs, late-passage MSCs were more sensitive to oxidative stress, exhibiting excessive ROS production and downregulating the expression of antioxidant proteins such as Cu/Zn-SOD, glutathione peroxidase (GPX), and catalase (CAT). This imbalance could impair cellular function and induce apoptosis, ultimately compromising MSCs quality for therapeutic use 58.

### 3.2 Tri-lineage differentiation

#### 3.2.1 MSCs differentiation

According to the standard definition, MSCs possess differentiation potential toward multiple cell types under specific culture conditions, including adipocytes, osteoblasts, chondroblasts, islet-like cells, hepatocyte-like cells, neural-like cells, and skeletal myocytes. This differentiation capacity confers significant therapeutic efficacy for regenerative medicine, as MSCs can migrate to tissue injury sites and differentiate into organ-specific cell types.

#### 3.2.2 Tri-lineage differentiation fates

Notably, senescent MSCs can affect their capacity for tri-lineage differentiation and differentiation direction, as confirmed by both common histological staining techniques and gene expression analysis. For example, using widely applied techniques, osteogenic differentiation declines, as indicated by decreased mineral deposition in Alizarin red-S staining and, particularly, downregulation of osteoblast markers such as alkaline phosphatase (ALP) and osteocalcin (OCN) 15,17,20,28,35,50,53,56,59,60.

Impaired chondrogenic lineage is evidenced by decreased sulfated proteoglycans in the cartilage matrix, stained with Alcian blue, and reduced gene expression levels of collagen type II (Col-II) 15,20,56,57,59,61.

While adipogenic differentiation potential is retained or even enhanced with aging 15,17,53,56,60, prolonged culture leads to a decline in adipogenic differentiation in MSCs undergoing *in vitro* senescence 28,35,50. This decline is primarily evidenced by lipid droplet accumulation, revealed through Oil Red O staining, and downregulation of adipogenic markers, including peroxisome proliferator-activated receptor gamma (PPARγ) and lipoprotein lipase (LPL) gene expression in senescent MSCs.

However, some evidence suggests that age has no significant effect on the tri-lineage differentiation of MSCs 62,63. One key reason for these contradictory findings is that MSCs are a heterogeneous population, meaning that MSCs from different donors or subgroups may yield different results 64. Table 3 summarizes the changes in tri-lineage differentiation direction of MSCs during *in vivo* and *in vitro* senescence.

## 4. Immunomodulatory Functions

### 4.1 Immune cells polarization status

It is well established that restoring the normal immunomodulatory function of immune cells is crucial for improving treatment outcomes in various age-related inflammatory diseases 65. As promising modulators of the immune system and immune responses, MSCs regulate both the innate and adaptive immune responses through direct cell contact with immune cells and paracrine effects. These include mediating the polarization of pro-inflammatory M1-like macrophages (M1) to anti-inflammatory M2-like macrophages (M2) and T helper 1 (Th1) and Th17 cells to regulatory T cells (Tregs), promoting the resolution of inflammation, cell and tissue regeneration, and damage repair 66-68.

Indeed, our recent study adds to the growing body of evidence that ADSCs contribute to the immunosuppressive effects in colorectal cancer patients with severe COVID-19, suggesting that interventions involving ADSCs could be a beneficial approach for patients who fail to respond to conventional therapies 69.

### 4.2 MSC2 to MSC1

With aging, MSCs derived from elderly donors undergo a shift from the anti-inflammatory MSC2 phenotype to the pro-inflammatory MSC1 phenotype, which triggers early immune and inflammatory responses in tissues. This shift promotes the activation and proliferation of effector T cells, favors the expansion and differentiation of Th1 and Th17 cells, and inhibits Tregs. This process is driven by the downregulation of intracellular proteins, including indoleamine 2,3-dioxygenase (IDO), prostaglandin E2 (PGE2), and programmed cell death 1 ligand 1 (PD-L1), as well as a decrease in factors such as transforming growth factor β (TGF-β) and IL-10 70. The downregulation of PD-L1 was observed during doxorubicin- or H2O2-induced UCB-MSCs senescence and in late-passaged UCB-MSCs. After the addition of an anti-PD-L1 blocker, the ability to inhibit T cell activation was significantly reduced, suggesting that the reduction in PD-L1 expression may be a key factor in the decline of their immunomodulatory capacity 71. Monitoring PD-L1 expression levels could provide a rapid assay for MSC-mediated immune suppression, which would align with the cell manufacturing process. Other studies of replicative-induced senescent MSCs have reported similar findings 31, indicating that the transformation of MSCs to the MSC1 phenotype is crucial in the development of senescence-associated immunomodulatory dysfunction and pro-aging activities.

### 4.3 Cellular microenvironment

Furthermore, senescent MSCs can alter the cellular microenvironment by secreting SASP factors such as chemokine ligand 2 (CCL2), tumor necrosis factor-α (TNF-α), interferon-inducible protein 10 (IP10), IL-6, and interferon-gamma (IFN-γ) in a paracrine manner. This contributes to the differentiation of macrophages into the M1-like polarization state, enhancing the inflammatory phase 72.

## 5. Mechanisms of MSC Senescence

**Figure 2:**
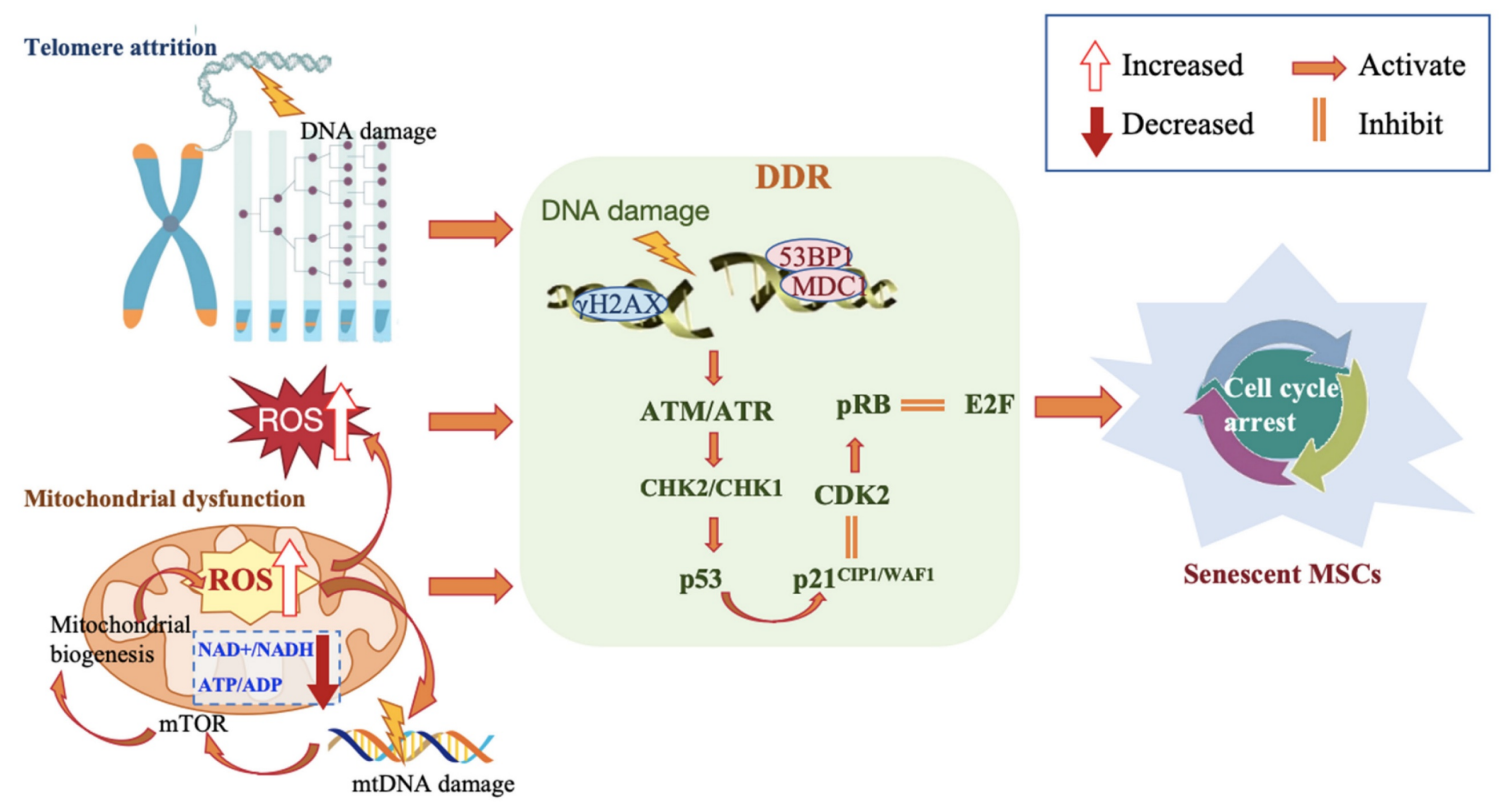
Molecular mechanisms associated with MSC senescence.

Driving MSCs senescence involves various interrelated molecular mechanisms, triggered by DNA damage, telomere attrition, reactive oxygen species (ROS), and mitochondrial dysfunction. These lead to progressive impairment or loss of self-renewal capacity and functional exhaustion, ultimately accelerating cell senescence (**Figure 2**).

### 5.1 DNA Damage

DNA damage contributes to persistent replicative senescence and the natural aging of MSCs. Continuous DNA damage accumulation results in reduced expression of stemness-related genes, disrupts transcriptional processes, and activates the DNA damage response (DDR) signaling pathway. Activation of DDR halts the cell cycle, inducing cellular senescence and progressive functional deterioration 73,74.

#### 5.1.1 DDR associated molecules

p16, phosphorylated-p53, and p21^Cip1/Waf1^ are DDR-associated molecules found to be upregulated in late-passaged MSCs 74. The p53 binding protein 1 (53BP1), phosphorylated histone H2AX (γH2AX), and the ATM pathway are key components of the DNA damage response and have been used to assess DNA damage 12. Gnani et al. reported a significant increase in the percentage of DDR marker 53BP1 foci-positive cells in MSCs derived from aged donors, along with activation of the phosphorylated form of the upstream DDR kinase ATM and the double-strand breaks (DSBs) marker γH2AX in MSCs 27. Similarly, cardiac MSCs from aged mice (20 months ± 2.55) also exhibited accumulated DNA damage, as evidenced by an increased percentage of γH2AX-positive cells 21. Evidence suggests that MSCs' ability to recognize endogenous and radiation-induced DNA double-strand breaks (DSBs) declines during long-term culture, characterized by a decrease in the number of γH2AX/53BP1 DSB repair foci and a gradual loss of ATM pathway activity 75.

#### 5.1.2 DNA repair mechanisms

Additionally, DNA repair pathways can recognize DNA damage and restore DNA integrity in MSCs through various mechanisms, including base excision repair (BER), nucleotide excision repair (NER), and DNA mismatch repair (MMR) 76. DNA repair deficiency increases the accumulation of DNA damage, leading to suppression of MSCs growth and self-renewal capacity, ultimately driving cell fate toward DNA damage-related senescence both *in vitro* and *in vivo*.

### 5.2 Telomere attrition

#### 5.2.1 Telomere attrition

MSCs senescence is closely associated with the loss of telomere length maintenance. Progressive or replicative telomere shortening is a major contributor to DNA damage, which exacerbates cell cycle arrest, apoptosis, differentiation bias or loss, and impaired replicative capacity of MSCs 77. Telomere attrition triggers the formation of telomere-associated DDR foci (TAFs) or telomere-induced DNA damage foci (TIFs), leading to overexpression of p53, p16, and p21, and attenuates mitochondrial metabolic activity through the peroxisome proliferator-activated receptor gamma co-activators 1α/β (PGC-1α/β). This, in turn, forcibly inhibits cell proliferation, sustains a persistent DNA damage signal, and increases MSCs susceptibility to senescence, even driving apoptosis or autophagy 78,79.

#### 5.2.2 POT1a

Protection of telomeres 1a (POT1a), a telomere-binding protein, has been found to maintain telomere length and genomic integrity. Recently, Nakashima et al. reported that POT1a-deficient MSCs led to the intracellular accumulation of excessive ROS and DNA damage, favoring adipogenesis over osteogenesis, which resulted in skeletal retardation in MSC-specific POT1a-deficient mice 80.

#### 5.2.3 TERT

Notably, telomerase reverse transcriptase (TERT) is the key determinant of telomerase activity, which is essential for maintaining telomere length. Telomerase deficiency accelerates telomere shortening, significantly reducing migratory and multi-differentiation capabilities, even in early-passaged MSCs 81,82. One study showed that overexpression of TERT enabled ADSCs to resist DNA damage accumulation during long-term culture and increased their tolerance to oxidative stress, thereby activating telomerase activity and preserving MSC biological functions 83.

### 5.3 ROS and mitochondrial dysfunction

Mitochondrial dysfunction is a hallmark and therapeutic target of the MSCs senescence process, driven by a series of pathological changes, including an increase in mitochondrial DNA (mtDNA) mutations, endogenous ROS accumulation, and ATP depletion. These factors contribute to the acceleration of senescence-associated DNA damage, ultimately impairing cellular functions 84.

#### 5.3.1 ROS dysfunction

The study reported that normal physiological ROS production was essential and beneficial for MSCs self-renewal. However, when unregulated intracellular ROS levels, particularly from mitochondria, increase dramatically and antioxidant defenses are significantly impaired, it is considered one of the mechanisms underlying replicative senescence in MSCs. This weakens self-renewal, proliferation, and osteogenic differentiation capabilities, induces cellular dysfunction and apoptosis, and promotes and exacerbates inflammation, leading to a stagnation in repopulating capacity 85,86. One example of its impact is that excessive production of intracellular ROS during aging can trigger DDR, which activates complex I of the mammalian target of rapamycin (mTORC1), leading to mitochondrial biogenesis and further increasing the generation of harmful ROS 87.

#### 5.3.2 Mitochondrial dysfunction

Researchers reported that senescent MSCs exhibited downregulation in the expression of mitochondrial biogenesis genes, such as peroxisome proliferator-activated receptor gamma coactivator-1 alpha (PGC-1α), SIRT, mammalian target of rapamycin (mTOR), and adenosine 5'-monophosphate (AMP)-activated protein kinase (AMPK). They also displayed lower mitochondrial membrane potential, reduced NAD^+^/NADH and ATP/ADP ratios 88, as well as altered mitochondrial morphology and bioenergetics 89, all of which were associated with MSCs senescence 90. Therefore, regulating mitochondrial function could rejuvenate senescent MSCs by photobiomodulation, altering their energy metabolism-related factors and pathways 91,92.

#### 5.3.3 Mtophagy and MT

Additionally, dysfunctional mitochondria are typically degraded through mitophagy, which protects cells from various cytotoxic stimuli and slows senescence in MSCs, thereby maintaining homeostasis and stemness. An overabundance of autophagy or genetic damage to the autophagy process can lead to the accumulation of dysfunctional organelles, promoting aging 93. Recent evidence has shown that harnessing the beneficial effects of intercellular mitochondrial transfer (MT) from MSCs can restore the bioenergetic profile and cell viability, increase mtDNA content, and reduce oxidative stress and inflammation 94, thereby delaying MSCs senescence or rejuvenating biological functions lost during this process.

## 6. Anti-Senescence Interventions

**Figure 3:**
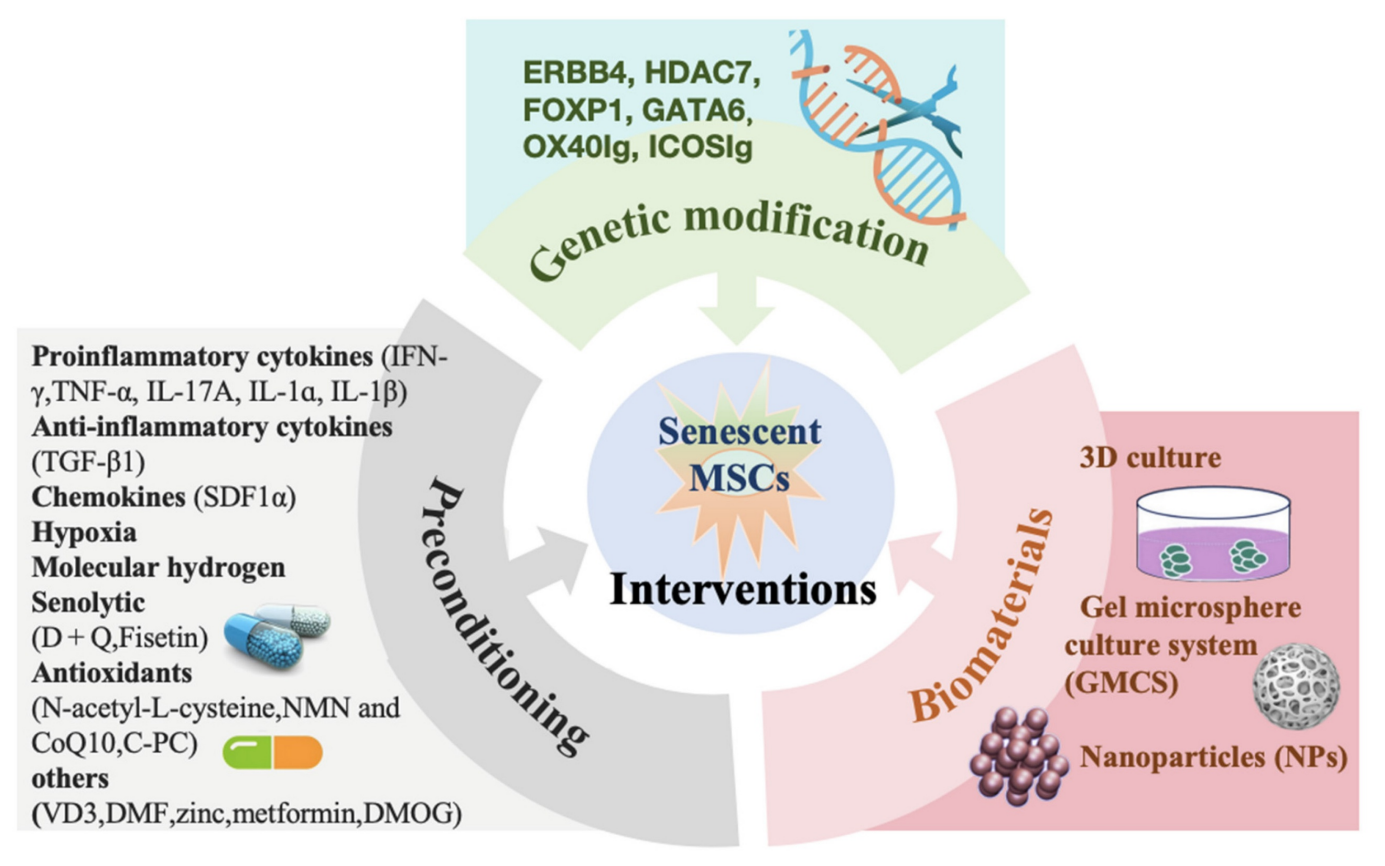
Rejuvenation interventions of senescent MSCs.

Given the challenges outlined above, various specific priming interventions have been proposed to enhance the functionality and therapeutic potential of MSCs through preconditioning, targeted genetic modification, and biomaterials. These interventions aim to repair self-renewal capacity and reshape the immunoregulatory and regenerative abilities of MSCs, ensuring optimal survival and function (**Figure 3**).

### 6.1 Preconditioning

#### 6.1.1 Inflammatory factor preconditioning

Preconditioning MSCs with specific or conditioned media containing proinflammatory cytokines before application is a widely used strategy to enhance the therapeutic effect of MSCs. This approach restores self-renewal capacity, increases *in vivo* cell survival, augments paracrine activity, and enhances tri-lineage differentiation capabilities of MSCs.

Specifically, preconditioning with IFN-γ has been reported to enhance the immunosuppressive phenotype, characterized by high IDO activity, and counteract the induction of paracrine senescence in cultured MSCs, alleviating the symptoms of graft-versus-host disease (GVHD) 95-97. Researchers designed an injectable synthetic hydrogel containing a biologically active form of IFN-γ, which induced strong immunomodulatory and anti-inflammatory capabilities in MSCs. This inhibited both activated T cell proliferation and monocyte differentiation into dendritic cells, accelerating the healing of mucosal wounds in a mouse colonic wound surgery model, and eliminating the need for *in vitro* manipulation 98. Additionally, TNF-α-preconditioned MSCs exhibited anti-inflammatory properties by reducing the capacity of peripheral immune cells to produce pro-inflammatory cytokines. This was accompanied by a decrease in low-density lipoprotein (LDL) levels and an elevation in high-density lipoprotein (HDL) levels, thereby suppressing systemic inflammation and reducing the size of atherosclerotic plaques 99. A recent study found that preconditioning ADSCs with IL-17A significantly enhanced their immunosuppressive responses on activated T cells and induced Treg expansion compared to naive MSCs, despite the similarity in their immunophenotype 100.

Notably, the use of cocktail treatments (IFN-γ synergized with TNF-α) to precondition MSCs can stimulate MSCs to synthesize abundant growth factors associated with wound repair and promote dermal fibroblast migration, proliferation, and activation, thus improving wound healing 101. Moreover, preconditioning with chemokines 106 and anti-inflammatory cytokines 107,108 can also prolong the effectiveness of therapy.

#### 6.1.2 Hypoxic preconditioning

Hypoxic preconditioning is another approach to reversing MSCs senescence, effectively enhancing MSCs biological functions, such as survival, proliferation, migration, and multilineage differentiation. This is achieved through activation of the PI3K/AKT-HIF-1α-CXCR4/CXCR7 pathway, upregulation of key cell cycle regulators (lncRNA SNHG16), and activation of the HIF-1α/Apelin/APJ axis 109,110. Furthermore, hypoxic preconditioning has been shown to significantly enhance the paracrine activity of MSCs, including increased expression of vascular endothelial growth factors, angiopoietin-1, erythropoietin, monocyte chemoattractant protein 1 (MCP-1), hepatocyte growth factor (HGF), and hypoxia-inducible factor-1α (HIF-1α), thereby improving the *in vitro* expansion of MSCs 109-111. Hypoxic preconditioning has also been shown to increase survival capacity, delay olfactory mucosa MSC (OM-MSC) senescence by upregulating miR-326/PTBP1/PI3K-mediated autophagy, and enhance neuroprotection, thereby augmenting therapeutic efficacy in intracerebral hemorrhage (ICH) 112.

In addition to hypoxic preconditioning, priming MSCs with molecular hydrogen has emerged as a promising approach to overcome cellular senescence. Molecular hydrogen preconditioning induces antioxidant, anti-inflammatory, and anti-apoptotic effects, primarily mediated by the ROS/p53/p21 signaling pathway, thereby improving therapeutic outcomes in a wide range of diseases 113.

#### 6.1.3 Senolytic drugs preconditioning

Given that senolytic drugs can effectively eliminate senescent cells, they have become a common approach to intervene in MSC senescence in recent years. For example, preconditioning MSCs with a senolytic mixture such as dasatinib (D) and quercetin (Q) has been shown to reduce the expression of senescence-related markers like p21 and p16, enhance mobilization and proliferation, and promote osteogenic differentiation of senescent BM-MSCs, resulting in bone organoids with restored bone remodeling 115. A recent study reported for the first time that the senolytic agent fisetin attenuated reactive oxygen species, SA-β-gal, and senescence-associated heterochromatin foci produced during the culture expansion of ADSCs, while maintaining the multilineage differentiation potential of amplified ADSCs 116.

Currently, the use of antioxidants offers a feasible strategy for MSCs rejuvenation. N-acetyl-L-cysteine 117, β-nicotinamide mononucleotide (NMN), and Coenzyme Q10 (CoQ10) 114 have been reported to maintain genome stability, telomerase activity, and telomere length, reduce TNF and IL-17 inflammatory signaling pathways, and enhance DNA repair even after prolonged expansion, thereby inhibiting MSCs senescence. A recent study by Liu et al. reported for the first time that C-phycocyanin (C-PC) could ameliorate MSCs senescence, specifically by restoring the proliferative activity of senescent MSCs to some extent, reducing harmful ROS production and aging-related phenotypes, and promoting adipogenic and osteogenic differentiation 119.

The development of additional drugs holds potential for effectively reversing MSC aging, enhancing therapeutic efficacy, and preventing age-related diseases. Examples include Vitamin D3 (VD3) 120, dimethyl fumarate (DMF) 121, zinc 122, metformin 123,124, and dimethyloxalylglycine (DMOG) 125. These drugs could induce ultrastructural and metabolic changes in MSCs, along with modifications in their secretome profiles, thereby delaying MSCs senescence. However, challenges remain in determining the optimal dosages, exposure duration, and managing potential therapeutic side effects. We summarize most priming preconditioning, as shown in Table 4.

### 6.2 Genetic modification

Over the past decade, various techniques for genetically modifying MSCs have been rapidly developed to enhance the secretion and expression of beneficial gene products, thereby imparting new characteristics to the cells and improving their therapeutic efficacy 126. For instance, MSCs genetically engineered to overexpress Erb-B2 receptor tyrosine kinase 4 (ERBB4) have been shown to reduce oxidative stress, decrease the senescent phenotype in vitro, and improve heart function by enhancing blood vessel density and reducing cardiac remodeling 127. Similarly, Li et al. demonstrated that MSCs overexpressing forkhead box P1 (FOXP1) played a crucial role in cell cycle regulation, enhancing MSCs self-renewal by suppressing p16INK4A activation 128. Further studies have shown that MSCs genetically engineered to overexpress both FOXP1 and histone deacetylase 7 (HDAC7) cooperate to promote MSCs replicative capacity, reduce cellular senescence, and enhance osteogenic potential *in vitro*, compared to MSCs overexpressing FOXP1 alone 129. Recently, MSCs transduced with either OX40Ig or ICOSIg have been reported to regulate MSCs function without altering their intrinsic characteristics and to alleviate various inflammatory diseases by blocking the OX40/OX40L or ICOS/ICOSL co-stimulatory pathways 130-132. In summary, gene overexpression imparts new characteristics to MSCs, leading to more potent targeted therapeutic effects.

In addition to gene overexpression, gene knockdown has also been shown to reverse MSCs senescence. Jiao et al. demonstrated that knockdown of the age-related pro-senescence factor GATA binding protein 6 (GATA6) significantly reduced the expression of p53 and p21CIP1, while downregulating the pro-inflammatory cytokines IL-6 and IL-1β, which are known to induce cellular senescence, in late-passaged MSCs, thereby mitigating MSCs senescence 133. Lin et al. further emphasized the significance of the GATA6/SOCS3/PDL1 pathway in regulating aging-associated changes in the immunomodulatory activity of MSCs 134.

Notably, Ruetz et al. 135 developed high-throughput *in vitro* and *in vivo* CRISPR-Cas9 screening platforms to identify genetic interventions that enhance the function of elderly neural stem cells, potentially delaying or reversing aging features and counteracting senescence in MSCs. To date, numerous technical challenges remain, including durability and safety concerns related to genetic engineering that still need to be addressed.

### 6.3 Biomaterials

Considering the biomimetic environment required for MSCs to maintain their properties and the targeted, controlled release of MSCs, advances in biomaterial engineering play a crucial role in improving the biophysical properties of MSCs, thereby enhancing their therapeutic efficacy. MSCs cultured using three-dimensional (3D) culture techniques or the gel microsphere culture system (GMCS) have been shown to enhance the integrated stress response of MSCs, preserving higher stemness, behaviors, and functionality compared to those cultured on conventional substrates 136,137. The 3D culturing system enhanced cell migration, proliferation, and multilineage differentiation capabilities 138, demonstrating superior bone regeneration in a bone defect model 139. Furthermore, this method improved therapeutic efficacy in treating inflammatory pulmonary diseases by enhancing the pulmonary delivery and *in vivo* survival of MSCs 140.

In recent years, nanoparticles (NPs) have played an increasingly important role as a biomaterial in supporting MSCs proliferation and stimulating multi-differentiation, ultimately enhancing tissue repair. For example, MSCs cultured on dentin disks embedded with silver nanoparticles (AgNPs) showed a significant increase in proliferation, migration, osteogenic differentiation, and improved wound repair and closure 141. Wang et al. developed an NAD^+^-dependent SIRT1-activated nanoplatform that dual-delivers resveratrol (RSV) and nicotinamide riboside (NR), which could delay MSCs senescence and promote bone formation 142.

Recently, Yang et al. loaded TNF-α-treated MSCs-derived exosomes onto micro/nano-network titanium (Ti) surfaces, which increased the secretion of anti-inflammatory cytokines by targeting the PI3K/AKT/mTOR pathway, and enhanced angiogenesis and osteogenesis through M2-macrophage polarization, thereby accelerating osseointegration in type 2 diabetes (T2D) conditions 143. Current research on the effects of applied mechanical forces in reducing MSCs senescence has shown that mechanical conditioning can rejuvenate senescing MSCs from elderly patients through oxidative stress and DNA damage repair mechanisms 144.

## 7. Conclusions

Numerous bench-to-bedside studies have shown that MSCs offer significant advantages. However, the potential negative impact of *in vivo* versus *ex vivo* senescence of MSCs must be carefully considered. The progressive senescence of MSCs not only leads to a gradual loss of cellular stemness but also causes broad immunomodulatory dysfunction, thereby hindering the therapeutic benefits for age-related diseases driven by senescent MSCs. Current anti-senescent interventions aim to rejuvenate senescing MSCs, restoring them from age-related decline to youthful vigor, preserving self-renewal capabilities, and reshaping their immunoregulatory and regenerative functions, while achieving a favorable safety/efficacy profile and minimizing side effects. Despite extensive research and efforts, each technique still has certain limitations and may vary between individuals. Therefore, from a treatment perspective, there is an urgent need to continue developing and optimizing novel approaches to reverse cellular senescence, thereby enhancing the therapeutic outcomes of off-the-shelf MSCs therapy for specific disorders while minimizing side effects. With advances in modern science, artificial intelligence (AI) could play a crucial role in MSC therapy, offering significant benefits to regenerative medicine in the future. The ultimate goal is to delay aging and combat the development of aging-related diseases, thereby improving health and extending longevity.

## Figures and Tables

**Table 1:** The surface phenotype associated with MSCs senescence

Species	Tissue source	Age (passage number)	Altered phenotype	Refs
Human	Adipose tissue	From early (p0) to late (p15)	CD105 ↓	20
	-	From early (p5) to late (p14)	CD106 ↓	22
	Bone marrow	From early (p4-6) to late	CD228 ↓	23
	Bone marrow	From young (20-40 years) to aged donors (45-60 years); From early (p3) to late (p14)	CD264 ↑	24
	Adipose tissue	From early (p5) to late (p15)	CD26 ↑	25
	Umbilical cord blood	From early (p4) to late (p10)	CD26 ↑	26
	Bone marrow	From young (<35 years) to aged donors (≥70 years)	CD146 ↓	27
	Bone marrow	From early (p2) to late (p10)	CD105 ↓, CD106 ↓	28
Mice	Myocardium	From young (4 months) to aged mice (20 months)	CD90 ↓	21

↑ increased; ↓ decreased.

**Table 2:** The composition of SASP released in senescent MSCs

Species	Tissue source	Age (passage number)	Variable factors	Refs
Human	Umbilical cord blood	From early (p4) to late (p10)	IL-6 ↑, IL-8 ↑, MCP-1 ↑, and CXCL5 ↑	26
Human	Umbilical cord blood	From early (p4-5) to late (more than p11)	MCP-1 ↑	42
Human	Umbilical cord blood	From early (p5) to late (p20)	IL-6 ↑, IGFBP-4 ↑, IGFBP-7 ↑ and MCP-1 ↑	43
Human	Bone marrow	From young (<35years) to aged donors (≥70 years)	IL6 ↑, MCP-1 ↑, IL8 ↑, IL-1α ↑, CXCL2 ↑	27
human	Bone marrow	From early (p2-5) to late (p9-14)	IL-1α ↑, and IL-8 ↑	30
Mouse	Bone marrow	From young (8 weeks) to aged (78 weeks); From early (p3) to late(p10)	IL-6 ↑, IL-10 ↓	35
Mouse	Bone marrow	From young (2 months) to aged (18 months)	IL-1α ↑, IL-5 ↑, IL-6 ↑, MCP-5 ↑	44

MCP-1, monocyte chemoattractant protein-1; CXCL5, C-X-C motif chemokine ligand 5; IGFBP, insulin-like growth factor binding protein; CXCL2, C-X-C motif chemokine ligand 2. ↑ increased; ↓ decreased.

**Table 3:** Changes in tri-lineage differentiation direction of MSCs *in vivo* and *in vitro* senescence

Species	Tissue source	Age (passage number)	Osteogenic differentiation	Chondrogenic differentiation	Adipogenic differentiation	Changes in specific transcription factor	Refs
*In vivo*							
Human	Adipose tissue	From young (>20 years) to aged (>70 years)	↓	↓	↑	Osteogenic: BMP-2 ↓, OPN ↓, OCN ↓; Chondrogenic: Col-II ↓; Adipogenic: PPAR-γ ↑	15
Mice	Bone marrow	From young (5 weeks) to aged (50 weeks)	↓	-	↑	Osteogenic: ALP ↓, OCN ↓; Adipogenic: PPAR-γ ↑, LPL ↑	53
Human	Adipose tissue	From young (2-6 years) to aged ( > 50 years)	↓	-	No differences	Osteogenic: OPN ↓	17
Human	Adipose tissue	From young ( <30 years) to aged (>60 years)	↓	↓	No differences	Osteogenic: OCN ↓, ALP ↓; Chondrogenic: ACAN ↓, Col-II ↓	56
Human	Adipose tissue	Infant Adult	↑	↓	↓	Osteogenic: OCN ↑, RUNX2 ↑; Chondrogenic: SOX9 ↓, Col-II ↓, COL10 ↓; Adipogenic:PPARγ ↓, LPL ↓	57
Horses	Bone marrow	From young (0 days) to aged (≥ 22 years)	↓	↓	-	Osteogenic: RUNX2 ↓; Chondrogenic: COMP↓ , ACAN ↓, MIA ↓, COL11A1 ↓	59
Rat	Bone marrow	From young (1-month) to aged (21-month)	↓	-	↑	Osteogenic: RUNX2 ↓, OSX ↓, ALP ↓, BSP ↓, OPN ↓, OCN ↓; Adipogenic: PPAR-γ ↑, aP2 ↑, RTN ↑	60
Human	Bone marrow	From young (18-49 years) to aged (≥50 years)	No differences	No differences	No differences	-	62
Human	Adipose tissue	From young (≤ 35 years) to aged (≥ 55 years)	No differences	-	No differences	-	63
*In vitro*							
Human	Adipose tissue	P0-P15	↓	↓	No differences	-	20
Human	Bone marrow	From early (p2) to late(p10)	↓	-	↓	Osteogenic: RUNX2↓; Adipogenic: PPAR-γ↓	28
Rat	Bone marrow	From early (p3) to late (p10)	↓	-	↓	Osteogenic: OCN ↓, ALP ↓, RUNX2 ↓, OSX ↓; Adipogenic: PPAR-γ ↓, Fabp4 ↓, adiponectin ↓, perilipin A ↓	35
Human	Bone marrow	From early (p4) to late (p12)	↓	-	↓	-	50
Human	Tonsil	From early (p3) to late (p15)	p3-p10 ↑; p10-p15 ↓	↓	↓	Osteogenic: OCN ↓ (after P10)	61

BMP-2, bone morphogenetic protein 2; OPN, osteopontin; OCN, osteocalcin; Col-II, collagen types II; PPAR-γ, peroxisome proliferator-activated receptor γ; ALP, alkaline phosphatase; LPL, lipoprotein lipase; ACAN, aggrecan core protein; RUNX, runt-related transcription factor 1; SOX9, sex determining region Y box protein 9; COL10, recombinant collagen type X; COMP, cartilage oligomeric matrix protein; MIA, cartilage-derived retinoic acid-sensitive protein; COL11A1, recombinant collagen type XI alpha 1 (COL11a1); OSX, osterix; BSP, bone sialoprotein; aP2, adipocyte protein 2; RTN, resistin; Fabp4, fatty acid binding protein 4. ↑ increased or upregulated; ↓ decreased or down-regulated.

**Table 4:** Multiple preconditioning regulating MSC senescence

Preconditioning	Model / tissue source	Effect	Mechanism	Disease	Refs
**Proinflammatory cytokines**					
IFN-γ	Mouse	The symptoms of GVHD in NOD-SCID mice ↓	Induction of IDO expression in MSCs via the IFN-γ-JAK-STAT1 pathway	GVHD	97
IFN-γ	Mouse/ Bone marrow	MSCs immunomodulatory function ↑, colonic mucosal wound healing rate ↑	IDO and PD-L1 expression ↑	Colonic wound	98
TNF-α	Mouse/ Bone marrow	Total cholesterol and LDL levels ↓, TNF-α and IFN-γ ↓, the spleens' weights ↓; HDL levels ↑, IL-10 ↑;	Tregs and Th1 ↑	Atherosclerosis	99
	Bone marrow	Chondrogenic differentiation ↑	Modulating SOX11 levels and WNT/β-catenin signaling	-	102
IL-17A	Human / Adipose tissue	The inhibitory rate of lymphocyte proliferation increased gradually	Tregs ↓	-	100
IL-1ɑ	Mouse / Bone marrow	Lesion volume ↓, infarct volume ↓, neurological deficits ↓; CBF in the ipsilateral hemisphere ↑	-	Ischemic stroke	103
IL-1β	Human / Umbilical cord blood	Migration ↑	Matrix metalloproteinase-3 via ERK1/2 pathway ↑	-	104
IFN-γ + TNF-α	Umbilical cord blood	proliferation, migration and activation of dermal fibroblasts ↑, type III collagen expression ↑, wound healing ↑	Expression of PDGF-BB via the PI3K/Akt signaling pathway ↑	-	101
IFN-γ + IL-1α, IFN-γ + IL-1β, IFN-γ + TNF-α	Human / Bone marrow	PBMC proliferation ↓	-	Parkinson's disease	105
**Chemokines**					
SDF1α	Rat / Bone marrow	MSCs survival ↑, cardiac function ↑, the peri-infarct capillary density ↑; reduced scar size ↓	Lactate dehydrogenase release ↓	Infarcted Myocardium	106
**Anti-inflammatory cytokines**					
TGF-β1	Mouse	MSCs ability to suppress T cell proliferation ↑, hepatic homing ↑, anti-inflammatory efficacy ↑	Surface expression of CXCR3 ↑	Acute carbon tetrachloride	107
	Rat / Umbilical cord blood	MSC proliferation and survival rate ↑; LPS-induced systemic injury ↓	RhoA expression ↑, phosphorylation of SMAD3 ↑	Lipopolysaccharide-induced acute lung injury	108
**Hypoxia**					
	Placenta-derived	proliferation capacity ↑; cell death and senescence ↓	PI3K/AKT pathway ↑	-	109
	Mouse / Bone marrow	New vessel formation during skeletal muscle reconstruction ↑	the level of VEGF and SDF-1 expression ↑	Muscle injury	110
	Mouse / Umbilical cord blood	Bone fracture healing ↑, MSCs proliferation and migration ability ↑, tube formation in umbilical vein endothelial cell ↑	Production of exosomal miR-126 through the activation of HIF-1α ↑	Bone fracture	111
	Mouse / Olfactory mucosa	MSCs senescence ↓; neuroprotection following intracerebral hemorrhage ↑	MiR-326/PTBP1/PI3K-mediated autophagy ↑	Intracerebral hemorrhage	112
	Olfactory mucosa	Angiogenesis-stimulatory activity of HBMECs ↑	MiR-612-TP53-HIF-1α-VEGF axis↑	-	114
**Senolytics**					
Dasatinib+ Quercetin (D + Q)	Mouse / Bone marrow	SA-β-gal^+^ cells ↓, p21 ↓, p16 ↓, IL6 ↓; proliferation rate↑, in vitro and in vivo osteogenic capacity ↑	Clearance of senescent cells	Calvarial defect	114
Fisetin	Human adipose tissue	ROS ↓, SA-β-gal ↓, senescence-associated heterochromatin foci ↓	-	-	116
**Antioxidants**					
N-acetyl-L-cysteine	Human bone marrow	Proliferation rate and multiple linage differentiation ability ↑; retains BM-MSCs with stable chromosome, DNA, telomere length, and telomerase activity	-	-	117
NMN and CoQ10	Human umbilical cord blood	H2O2-induced senescence ↓, TNFα mRNA level ↓; proliferation migration ability ↑	DNA repair ability↑, cyclin expression ↑; TNF and IL-17 inflammatory signaling pathways ↓	-	118
C-PC	Rat / Adipose tissue	Proliferation ↑, adipogenic and osteogenic differentiation ↑, anti-inflammatory factors ↑, SOD activity ↑; ROS or MDA ↓,	ZDHHC5-Mediated Autophagy via PI3K/AKT/mTOR Pathway	D-Gal-induced accelerated aging	119
**Other drugs**					
VD3	Human bone marrow	Proliferation ↑, osteogenic differentiation ↑; replicative senescence ↓	SIRT1-FoxO3 signaling pathways ↑	-	120
DMF	Rat / Adipose tissue	Survivability ↑, proliferation and antioxidant capability ↑, neuroprotective effect ↑; restore impaired learning and memory	Nrf2 ↑	Alzheimer's disease	121
Zinc	Human umbilical cord blood	Cell adhesion, migration and self-renewal potential ↑	-	-	122
Metformin	Human bone marrow	Percentage of SA-β-Gal-positive cells ↓, the levels of p53, p21, and p16 ↓.	Autophagy by activating the AMPK pathway ↑	-	123
	Human dental pulp	Percentage of SA-β-gal-positive cells ↓, the levels of p53, p21, and p16 ↓	Targeting CAB39 through the AMPK/mTOR signaling pathway	-	124
DMOG	Human umbilical cord blood	Migratory and proliferative capacities ↑; altered ultrastructural morphology	-	-	125

GVHD, graft-versus-host disease; IDO, intracellular protein indoleamine 2,3-dioxygenase; JAK-STAT, janus kinase - signal transducer and activator of transcription; PD-L1, rogrammed cell death 1 ligand 1; LDL, low-density lipoprotein; HDL, high-density lipoprotein; SOX11, SRY-box transcription factor 11; CBF, cerebral blood flow; ERK, extracellular regulated protein kinases; PDGF-BB, platelet-derived growth factor-BB; PI3K/Akt, phosphatidylinositol 3-kinase / protein kinase B; PBMC, peripheral blood mononuclear cell; SDF1α, stromal cellderived factor 1α; CXCR3, chemokine C-X-C-motif receptor 3; SMAD3, mothers against DPP homolog 3; VEGF, vascular endothelial growth factor; HIF-1α, hypoxia inducible factor-1α; PTBP1, polypyrimidine tract binding protein 1; ROS, reactive oxygen species; NMN, β-nicotinamide mononucleotide; CoQ10, Coenzyme Q10; C-PC, C-phycocyanin; SOD, superoxide dismutase; MDA, malondialdehyde; VD3, Vitamin D3; SIRT1, Sirtuin 1; FoxO3, forkhead box O3; DMF, dimethyl fumarate; Nrf2, nuclear factor erythroid 2-related factor 2; AMPK, adenosine 5'-monophosphate-activated protein kinase; CAB39, calcium binding protein 39; DMOG, dimethyloxalylglycine. ↑ increased, upregulated or enhanced; ↓ decreased, down-regulated or impaired.‌‌‌
